# Double Sampling with Multiple Imputation to Answer Large Sample Meta-Research Questions: Introduction and Illustration by Evaluating Adherence to Two Simple CONSORT Guidelines

**DOI:** 10.3389/fnut.2015.00006

**Published:** 2015-03-09

**Authors:** Patrice L. Capers, Andrew W. Brown, John A. Dawson, David B. Allison

**Affiliations:** ^1^Office of Energetics and Nutrition Obesity Research Center, School of Public Health, University of Alabama at Birmingham, Birmingham, AL, USA; ^2^Section on Statistical Genetics, University of Alabama at Birmingham, Birmingham, AL, USA; ^3^Department of Nutrition Sciences, University of Alabama at Birmingham, Birmingham, AL, USA; ^4^Department of Biostatistics, University of Alabama at Birmingham, Birmingham, AL, USA

**Keywords:** double sampling, multiple imputation, CONSORT, meta-research, adherence, modeling

## Abstract

**Background:** Meta-research can involve manual retrieval and evaluation of research, which is resource intensive. Creation of high throughput methods (e.g., search heuristics, crowdsourcing) has improved feasibility of large meta-research questions, but possibly at the cost of accuracy.

**Objective:** To evaluate the use of double sampling combined with multiple imputation (DS + MI) to address meta-research questions, using as an example adherence of PubMed entries to two simple consolidated standards of reporting trials guidelines for titles and abstracts.

**Methods:** For the DS large sample, we retrieved all PubMed entries satisfying the filters: RCT, human, abstract available, and English language (*n* = 322, 107). For the DS subsample, we randomly sampled 500 entries from the large sample. The large sample was evaluated with a lower rigor, higher throughput (R_LO_T_HI_) method using search heuristics, while the subsample was evaluated using a higher rigor, lower throughput (R_HI_T_LO_) human rating method. Multiple imputation of the missing-completely at-random R_HI_T_LO_ data for the large sample was informed by: R_HI_T_LO_ data from the subsample; R_LO_T_HI_ data from the large sample; whether a study was an RCT; and country and year of publication.

**Results:** The R_HI_T_LO_ and R_LO_T_HI_ methods in the subsample largely agreed (phi coefficients: title = 1.00, abstract = 0.92). Compliance with abstract and title criteria has increased over time, with non-US countries improving more rapidly. DS + MI logistic regression estimates were more precise than subsample estimates (e.g., 95% CI for change in title and abstract compliance by year: subsample R_HI_T_LO_ 1.050–1.174 vs. DS + MI 1.082–1.151). As evidence of improved accuracy, DS + MI coefficient estimates were closer to R_HI_T_LO_ than the large sample R_LO_T_HI_.

**Conclusion:** Our results support our hypothesis that DS + MI would result in improved precision and accuracy. This method is flexible and may provide a practical way to examine large corpora of literature.

## Introduction

Meta-research, or “research on research,” describes investigations of research itself, including describing how research is conducted and reported, and aggregating and rating studies such as in meta-analyses. However, manually retrieving and analyzing the vast archives of written material to evaluate meta-research questions can be very time consuming and costly. The resource-intensity of this process leads many researchers to narrow the scope of inquiry in some way to answer questions of interest, which might, in principle, be explored more broadly. For example, Kaiser et al. (1) explored only top-tier nutrition and obesity journals to examine the quality of randomized controlled trials (RCTs) by funding source, and Kiriakou et al. (2) examined the quality of abstracts according to the consolidated standards of reporting trials (CONSORT) guidelines by only reviewing leading journals in oral implantology. Recent literature has suggested several strategies to minimize the time and cost involved in meta-research. These strategies include using PubMed medical subject headings (MeSH) terms and pre-built dictionaries (dictionaries created to identify key elements of studies, e.g., population, design) to identify characteristics of abstracts of interest (3), or evaluating larger corpora of literature using crowdsourcing (4). While these strategies provide useful information, a modeling approach incorporating *both* the resource-intensive evaluation of articles by humans *and* the efficient evaluation of articles using search heuristics may enable a researcher to capture a larger scope of articles in a straightforward way to answer a question at hand, yet retain the benefits of rigorous human coding.

One potential approach employs double sampling, where two samples are taken: a large sample and a subsample (from the large sample). The appropriate sample size of the subsample “depends on the relative costs of observing the two variables and on the strength of the ratio relationship between them [Ref. (5), p.160],” with precedents and guideposts available to help determine an appropriate sample size [e.g., Ref. (6)]. Given a concretely specified portion of the scientific literature, we can take a subsample wherein we evaluate the characteristics of interest using resource-intensive, human-based rating (e.g., manual retrieval and analysis) to create higher rigor, lower throughput (R_HI_T_LO_) data. In this subsample, we can also evaluate the characteristics of interest using a less-resource intensive, but often also less accurate (e.g., search heuristics), method to create lower rigor, higher throughput (R_LO_T_HI_) data, and then compare these two values.

**Figure 1:**
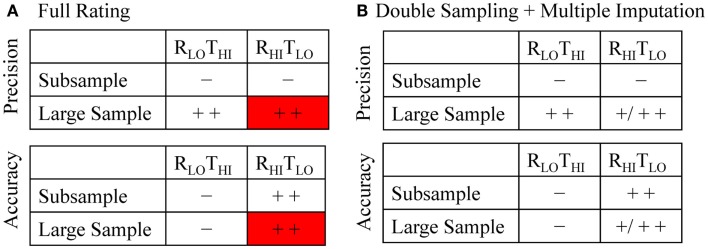
**Illustration of the precision and accuracy expected in the full rating (A) and the double sampling with multiple imputation (B) of titles and abstracts**. Minus signs indicate low precision or accuracy and the plus sign indicate precision or accuracy (single – improved, double – high). **(A)** Both methods are expected to be more precise in the large sample whereas the R_HI_T_LO_ method is expected to have increased accuracy in both samples. The red box indicates the desired, but impractical, R_HI_T_LO_ evaluation of a large dataset. **(B)** Both methods are expected to be more precise in the large sample whereas the R_HI_T_LO_ method is expected to have high accuracy in the subsample and increased accuracy in the large sample. R_LO_T_HI_, lower rigor, higher throughput; R_HI_T_LO_, higher rigor, lower throughput.

**Figure 2:**
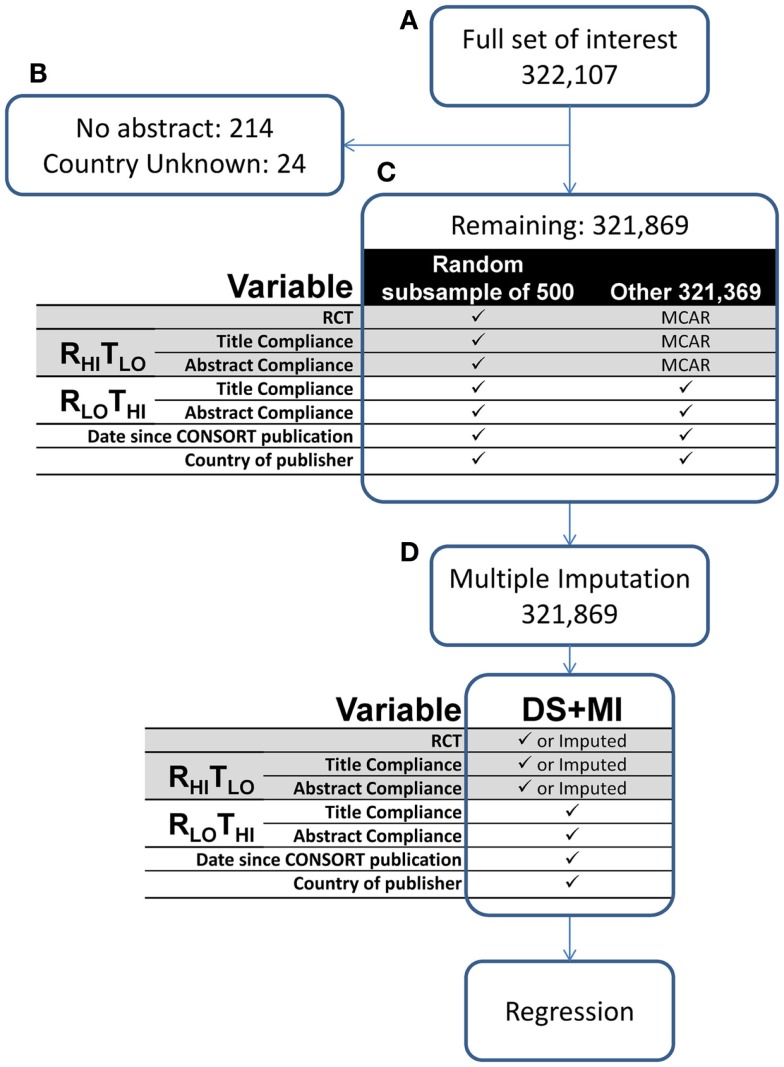
**Flow of PubMed entry selection, ratings, and statistical analysis**. **(A)** All possible human RCTs in the English language with abstract from PubMed were identified. **(B)** All articles without abstract or country identification were excluded. **(C)** From the remaining articles, a subsample was randomly selected and the R_HI_T_LO_ method was applied. The R_LO_T_HI_ method was applied to both the subsample and large sample to collect the variables of interest as well as auxiliary information (date since CONSORT and country publisher). The remaining data of the large sample are MCAR. **(D)** The available information was used to inform the imputation followed by statistical analysis. DS + MI, double sampling with multiple imputation; MCAR, missing completely at random; RCT, randomized controlled trial; R_HI_T_LO_, higher rigor, lower throughput; R_LO_T_HI_, lower rigor, higher throughput.

The two methods differ with respect to accuracy and precision depending on whether they are used on the subsample or the large sample (Figure 1A). Using the R_LO_T_HI_ on the subsample alone is quick and easy, but will likely have large random and possibly systematic errors (that is, low precision and likely inaccurate). Using the R_HI_T_LO_ method alone is not only more accurate but also more resource-intensive, which may not allow samples of sufficient size to offer the power and precision desired. The ability to apply the R_LO_T_HI_ method to the large sample improves precision compared to the results of the subsample alone, but is still likely prone to systematic error. To avoid this problem, applying the R_HI_T_LO_ method to the large sample would be ideal. However, this is impractical to perform on large corpora of literature in a timely manner. Instead, we propose using the R_HI_T_LO_ of the subsample alongside the R_LO_T_HI_ for the large sample to estimate the R_HI_T_LO_ of the large sample (Figures 1B and 2).

Because the R_HI_T_LO_ values in the large sample are missing completely at random (MCAR, because the subsample is a random sample of the large sample), we can estimate the large sample R_HI_T_LO_ values via multiple imputation (5, 7, 8). A key point is that it is necessary that the random subsample and the rest of the large sample have similar characteristics, other than the fact that the former has an additional R_HI_T_LO_ measurement and the latter does not. If this assumption holds, then imputation of the R_HI_T_LO_ in the rest of the large sample will be valid regardless of how small (proportionally) the subsample is compared with the large sample. Randomization guarantees that as the size of the full sample and of the subsample increase, any discrepancies in either observed or unobserved characteristics between the full sample and the subsample due to chance will on average shrink toward zero chance differences between the two. Multiple imputation is an approach to filling in missing data in a systematic, unbiased manner based on other data available in the dataset. The inclusion of auxiliary variables that provide additional information, such as date of publication (DP), about the missing data can help either reduce the bias or increase the power (7) and help inform the imputation.

In this paper, we focus on a method employing double sampling with multiple imputation (DS + MI). The aim of the paper is to describe the method and illustrate its application through evaluating whether titles and abstracts adhere to two simple CONSORT guideline criteria. As shown schematically in Figure 1B, we expect that the precision of the large sample R_HI_T_LO_ estimates calculated through DS + MI will be higher than estimates on the subsample alone as a function of increased sample size; how precise the estimate is will depend on the amount of missing information that needs to be imputed, so at best the precision will be as high as the large sample R_LO_T_HI_ method. In terms of accuracy, we expect the DS + MI estimates to be more accurate than the large sample R_LO_T_HI_ estimates, with the accuracy dependent on the quality of the R_LO_T_HI_ and the amount of missing information that needs to be imputed. We therefore hypothesize that DS + MI will result in a more precise estimate of title and abstract compliance among countries and across time than the subsample R_HI_T_LO_ and more accurate than the large sample R_LO_T_HI_, without having to rate all of the entries manually.

## Materials and Methods

To investigate the utility of this technique, we explored two of the criteria outlined in the CONSORT guidelines. The CONSORT guidelines were created in 1996 in an effort to improve the quality of reporting for RCTs (9). These guidelines consist of 25 items, of which we investigated two simple items for the purpose of illustration: (a) whether the title stated that the study was a RCT; and (b) whether the abstract was structured (e.g., headings of Introduction, Methods, Results, Conclusion).

### Data

Our large sample was the entire PubMed database available as of July 28, 2014, subject to the following filters: RCTs, humans, English language, and abstract available (*n* = 322, 107; Figure 2A). Entries that had no abstract (*n* = 214, despite using the PubMed “abstract available” filter) or no country listed in the place of publication field (*n* = 24) were excluded (Figure 2B). From this we obtained a simple random sample (via pseudorandom number generator) of 500 entries as our subsample. The 500 entries were then independently rated (Patrice L. Capers and Andrew W. Brown) to establish the R_HI_T_LO_ data.

### Establishing R_HI_T_LO_ data

The subsample entries were rated on the following:

1. Did the title denote that the study was a RCT?
  CONSORT Item 1a. “Identification as a randomized trial in the title … Authors should use the word ‘randomised’ in the title to indicate that the participants were randomly assigned to their comparison groups.” (10)
2. Was the abstract structured?
  CONSORT Item 1b. “Structured summary of trial design, methods, results, and conclusions … We strongly recommend the use of structured abstracts for reporting randomised trials. They provide readers with information about the trial under a series of headings pertaining to the design, conduct, analysis, and interpretation.” [Ref. (10, 11) http://www.consort-statement.org/checklists/view/32-consort/67-abstract]
3. Was the study actually an RCT?
  For the purpose of this study we were interested in randomized controlled trials in humans, written in the English language, and that have an available abstract. Classification of RCT status was based on the R_HI_T_LO_ raters (Patrice L. Capers and Andrew W. Brown).

For articles where Patrice L. Capers and Andrew W. Brown were uncertain whether a title and abstract were from an RCT, we assumed that PubMed was correct in identifying the entry as an RCT. Any disagreements were resolved by consensus. If no consensus could be met on the title and abstract alone, we retrieved the full article for full review.

### Defining the R_LO_T_HI_ method

For title compliance, the R_LO_T_HI_ method for the title looked for the word “random,” including variants with any prefixes or suffixes, anywhere in the title. For abstract compliance, the R_LO_T_HI_ required that an abstract needed to contain words representative of at least three of four headings: Introduction, Methods, Results, and Conclusion. Understanding that a variety of descriptors are used for these subheadings (e.g., Problem or Background may be used in place of Introduction), we grouped a variety of words into the proper identifier categories (Table S1 in Supplementary Material). The R_LO_T_HI_ was subsequently applied to the entire large sample (including entries from the subsample).

### Additional meta-data

We also identified the country from which abstracts were published using the PubMed place of publication (PL) tag. We used this tag to categorize papers in the following manner, based on data from the CIA World Factbook [Ref. (12); Table S2 in Supplementary Material]: (1) the United States (US), (2) non-US but primarily English speaking countries (ESC), and (3) all other countries (NESC). DP was derived from the “DP” tag in PubMed. Five conditions were used with increasing granularity to code the DP: if multiple years are listed, only the first year was retained; when only years were listed, they were set to the first of the year; when only years and seasons were listed, they are set approximately to the equinoxes or solstices as appropriate (March 21, June 21, September 21, and December 21); when only years and months were listed, they were set to the first of the month; when a full date were available, it was used directly. The variable for publication year was centered on the publication date of CONSORT (August 28, 1996) to aid in the interpretation of the results.

### Multiple imputation

From the subsample we gathered information on the variables of interest that had not yet been determined in the large sample making the data MCAR (Figure 2C). The R_LO_T_HI_ method was utilized in both the subsample and large sample to extract information of the variables of interest as well as auxiliary information (date since CONSORT and country of publisher). Information from both methods was used to inform the imputation (Figure 2D). The data were imputed using the “mice” package (version 2.22) of R (version 3.0.1) with a fixed but randomly selected seed set to 418. A total of 20 imputations with 100 within-imputation iterations were computed.

### Statistical analysis

Higher rigor, lower throughput results were compared against the R_LO_T_HI_ results from the subsample using phi coefficients, calculated using the phi command of the R “psych” package (version 1.4.8.11). The Phi coefficient is equivalent to the Pearson product moment correlation coefficient of the dichotomous variables, and therefore provides a single number expressing the similarity between the two methods. Descriptive analyses of the R_HI_T_LO_ and R_LO_T_HI_ data are tabled as counts of publications unless otherwise specified.

Logistic regression was used to model title compliance, structured abstract compliance, and both together as a function of country, year, and the interaction of country and year. Because only 79.8% of abstracts were rated as RCTs in the subsample, we imputed a study’s RCT status, and analyses were limited only to abstracts that were imputed as RCTs. Logistic regressions for imputed data were calculated using the glm.mids extension (mice package, version 2.22), while the regressions for the subsample were calculated using glm in the base package (R version 3.0.1). We hypothesized that US and ESC would have similar levels of compliance, while NESC would have lower compliance but would be rapidly improving (i.e., a larger NESC-by-time interaction term). Comparisons among the R_LO_T_HI_ and R_HI_T_LO_ results from the subsample and the R_LO_T_HI_ and the DS + MI (as estimates for R_HI_T_LO_) results from the large sample were used to evaluate precision and accuracy. Logistic regression coefficients and confidence intervals are reported in exponentiated form (i.e., odds ratios and their 95% confidence intervals).

## Results and Discussion

Our PubMed search (using the filters: humans, RCTs, and English language with abstract) retrieved entries that did not have an abstract or a country of publication. However, this number was fairly small considering the amount of entries contained within the data set. In our subsample, 20% of the entries were not RCTs according to our guidelines. Our estimate for the percent of actual RCTs in the large sample based on our subsample was 79.8% [95% CI: (76.28, 83.32)]. While there was unequal representation of countries in the entries available, the distribution was similar between the large sample and the subsample (US 51 vs. 51%, ESC 29 vs. 30%, NESC 20 vs. 18%, respectively). If not otherwise stated, discussion of results is about DS + MI results.

### Compliant title

Out of the 500 entries that PubMed tagged as RCTs in humans, only 399 were found to be actual RCTs according to the R_HI_T_LO_ method. Excluding non-RCTs, 28% of the titles were compliant with CONSORT guidelines (Table 1). The R_LO_T_HI_ was able to categorize entries identically to the R_HI_T_LO_ given that studies were actually RCTs (phi coefficient = 1.00), but resulted in false positives when non-RCTs were included (phi coefficient = 0.96). The false positives in the R_LO_T_HI_ search arose from titles containing either the word “random” or “RCT” but the paper was actually a study protocol or secondary analysis of RCTs.

**Table 1:** **Subsample ratings of title compliance with CONSORT**.

	Full subsample				RCTs in subsample		
	R_LO_T_HI_ method				R_LO_T_HI_ method		
**R_HI_T_LO_ method**		Non-compliant	Compliant	**R_HI_T_LO_ method**		Non-compliant	Compliant
	Non-compliant	380	7		Non-compliant	289	0
	Compliant	0	113		Compliant	0	110
**Phi coefficient**	0.96			**Phi coefficient**	1.00		

**Figure 3:**
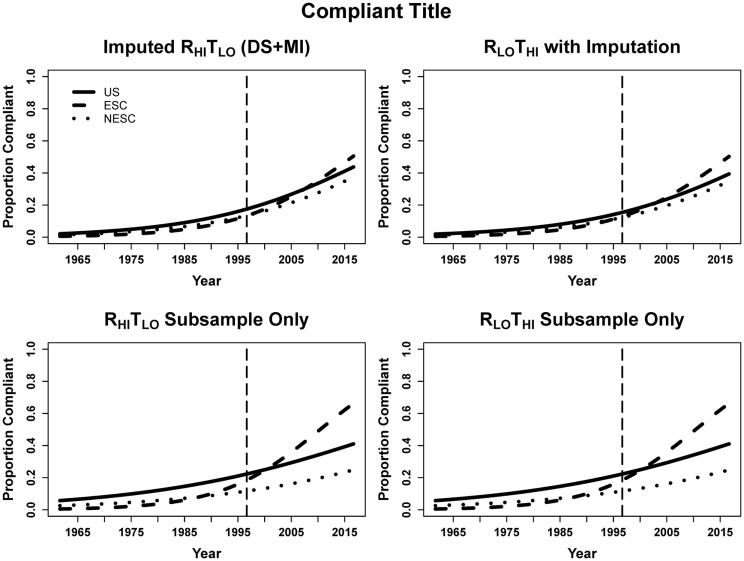
**Logistic curves for title compliance using imputation, the R_HI_T_LO_ and R_LO_T_HI_ for the large sample and subsample by year**. The vertical line represents the initiation of the CONSORT guidelines in 1996. US, United States; ESC, non-US but primarily English speaking countries; NESC, all other countries.

For every year beyond August 28, 1996 publications had 6.7% greater odds of being title compliant. ESC entries improved more rapidly overtime compared to US, as evidenced by the significant ESC-by-year term. The odds per year change for ESC and NESC are dependent on multiplying the year and the respective interaction terms, resulting in 9.9% greater odds per year for ESC, 7.0% for NESC, and 6.7% for US. In the subsample, entries published every year since CONSORT had 4.5% greater odds of being title complaint. Because of the low precision, no other predicators were significant in the subsample. Since the subsample R_LO_T_HI_ and R_HI_T_LO_ data are identical, the regressions on the subsample are identical (Table 2; Figure 3).

**Table 2:** Logistic regression of large sample and subsample for compliant titles*.

	R_HI_T_LO_ imputed (DS + MI)^a^	R_LO_T_HI_ with imputed RCT^b^	R_HI_T_LO_ subsample	R_LO_T_HI_ subsample
Intercept	0.212 (0.057, 0.787); *p* = 0.023	0.183 (0.029, 1.164); *p* = 0.069	0.287 (0.191, 0.431); *p* < 0.001	0.287 (0.191, 0.431); *p* < 0.001
ESC	0.726 (0.294, 1.794); *p* = 0.467	0.813 (0.383, 1.726); *p* = 0.572	0.793 (0.348, 1.806); *p* = 0.580	0.793 (0.348, 1.806); *p* = 0.580
NESC	0.733 (0.381, 1.407); *p* = 0.331	0.771 (0.500, 1.190); *p* = 0.226	0.460 (0.161, 1.311); *p* = 0.146	0.460 (0.161, 1.311); *p* = 0.146
Year	1.067 (1.021, 1.115); *p* = 0.006	1.065 (1.028, 1.105); *p* = 0.002	1.045 (1.005, 1.087); *p* = 0.026	1.045 (1.005, 1.087); *p* = 0.026
ESC-by-year	1.030 (1.003, 1.058); *p* = 0.034	1.033 (1.009, 1.058); *p* = 0.009	1.066 (0.988, 1.149); *p* = 0.098	1.066 (0.988, 1.149); *p* = 0.098
NESC-by-year	1.002 (0.983, 1.022); *p* = 0.800	1.003 (0.984, 1.023); *p* = 0.733	1.002 (0.914, 1.097); *p* = 0.973	1.002 (0.914, 1.097); *p* = 0.973

*All values are presented as: “odds ratios (95% CI); p value.”

*^a^The higher rigor, lower throughput (R_HI_T_LO_) imputed values represent the double sampling with multiple imputation (DS + MI) approach*.

*^b^Values in the lower rigor, higher throughput (R_LO_T_HI_) with imputed RCT column represent the results of the R_LO_T_HI_ method of the entries in the large sample determined to be RCT based on the imputation*.

*ESC, non-US but primarily English speaking countries; NESC, all other countries*.

### Compliant abstract

The consistency in ratings between the R_HI_T_LO_ and R_LO_T_HI_ methods was high (Table 3), but less than for title compliance. The R_HI_T_LO_ and R_LO_T_HI_ methods were positively correlated (phi coefficient = 0.92) for both the full subsample and only RCTs within the subsample. From the subsample, 51–53% of abstracts were compliant.

**Table 3:** **Subsample ratings of abstract compliance with CONSORT**.

	Full subsample				RCTs in subsample		
	R_LO_T_HI_ method				R_LO_T_HI_ method		
**R_HI_T_LO_ method**		Non-compliant	Compliant	**R_HI_T_LO_ method**		Non-compliant	Compliant
	Non-compliant	226	18		Non-compliant	172	14
	Compliant	2	254		Compliant	0	211
**Phi coefficient**	0.92			**Phi coefficient**	0.92		

**Figure 4:**
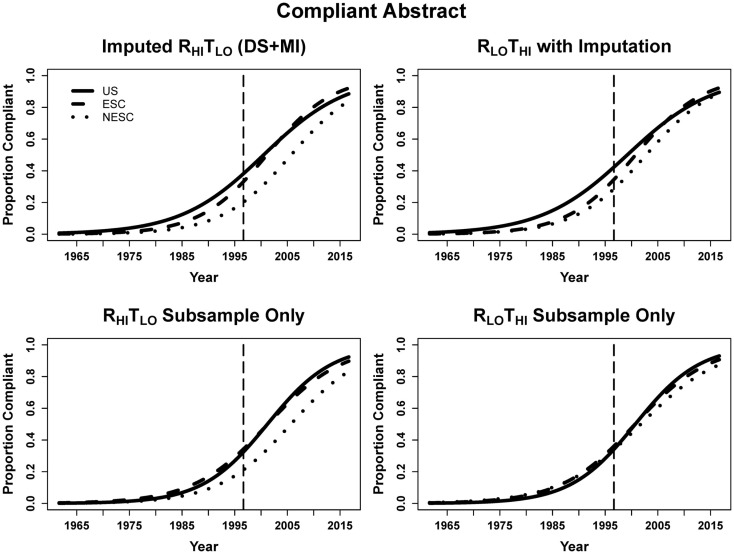
**Logistic curves for abstract compliance using imputation, the R_HI_T_LO_ and R_LO_T_HI_ for the large sample and subsample by year**. The vertical line represents the initiation of the CONSORT guidelines in 1996. US, United States; ESC, non-US but primarily English speaking countries; NESC, all other countries.

DS + MI estimated that for each year beyond CONSORT the odds of the abstract being structured was 13.5% greater using the R_HI_T_LO_ method. At the time of CONSORT publication, NESC abstracts were significantly less compliant than both the ESC and NESC. The significant interaction terms for ESC-by-year and NESC-by-year indicate that ESC and NESC were increasing the odds of abstract compliance faster than US (17.1% per year for ESC, 16.3% for NESC vs. 13.5% for US, calculated by multiplying the year and respective interaction terms). In the subsample, the larger error from the small sample size again resulted in only year being significant, with abstracts having 17.5 and 17.6% greater odds of being structured using the R_HI_T_LO_ and R_LO_T_HI_ methods, respectively, every year beyond August 1996 (Table 4; Figure 4).

**Table 4:** Logistic regression of large sample and subsample for compliant abstracts*.

	R_HI_T_LO_ imputed (DS + MI)^a^	R_LO_T_HI_ with imputed RCT^b^	R_HI_T_LO_ subsample	R_LO_T_HI_ subsample
Intercept	0.618 (0.473, 0.806); *p* = 0.001	0.733 (0.660, 0.815); *p* < 0.001	0.480 (0.310, 0.745); *p* = 0.001	0.518 (0.336, 0.798); *p* = 0.003
ESC	0.803 (0.635, 1.015); *p* = 0.065	0.718 (0.668, 0.771); *p* < 0.001	1.086 (0.509, 2.317); *p* = 0.830	1.091 (0.516, 2.305); *p* = 0.820
NESC	0.411 (0.277, 0.610); *p* < 0.001	0.545 (0.500, 0.595); *p* < 0.001	0.559 (0.211, 1.480); *p* = 0.242	0.993 (0.429, 2.297); *p* = 0.987
Year	1.135 (1.115, 1.154); *p* < 0.001	1.130 (1.121, 1.139); *p* < 0.001	1.175 (1.119, 1.233); *p* < 0.001	1.176 (1.121, 1.234); *p* < 0.001
ESC-by-year	1.032 (1.025, 1.039); *p* < 0.001	1.034 (1.029, 1.040); *p* < 0.001	0.980 (0.905, 1.060); *p* = 0.614	0.980 (0.906, 1.061); *p* = 0.620
NESC-by-year	1.026 (1.016, 1.035); *p* < 0.001	1.027 (1.020, 1.035); *p* < 0.001	0.985 (0.898, 1.080); *p* = 0.746	0.969 (0.890, 1.055); *p* = 0.463

*All values are presented as: “odds ratios (95% CI); p value.”

*^a^The higher rigor, lower throughput (R_HI_T_LO_) imputed values represent the double sampling with multiple imputation (DS + MI) approach*.

*^b^Values in the lower rigor, higher throughput (R_LO_T_HI_) with imputed RCT column represent the results of the R_LO_T_HI_ method of the entries in the large sample determined to be RCT based on the imputation*.

*ESC, non-US but primarily English speaking countries; NESC, all other countries*.

### Compliant title + abstract

When examining entries in the subsample where both the title and abstract were compliant, we found 17–21% to be in compliance. Ratings were similar between the R_HI_T_LO_ and R_LO_T_HI_ methods (phi coefficient = 0.96, 0.99; Table 5).

**Table 5:** **Subsample ratings of both title and abstract compliance with CONSORT**.

	Full subsample				RCTs in subsample		
	R_LO_T_HI_ method				R_LO_T_HI_ method		
**R_HI_T_LO_ method**		Non-compliant	Compliant	**R_HI_T_LO_ method**		Non-compliant	Compliant
	Non-compliant	408	6		Non-compliant	313	2
	Compliant	0	86		Compliant	0	84
**Phi coefficient**	0.96			**Phi coefficient**	0.99		

**Figure 5:**
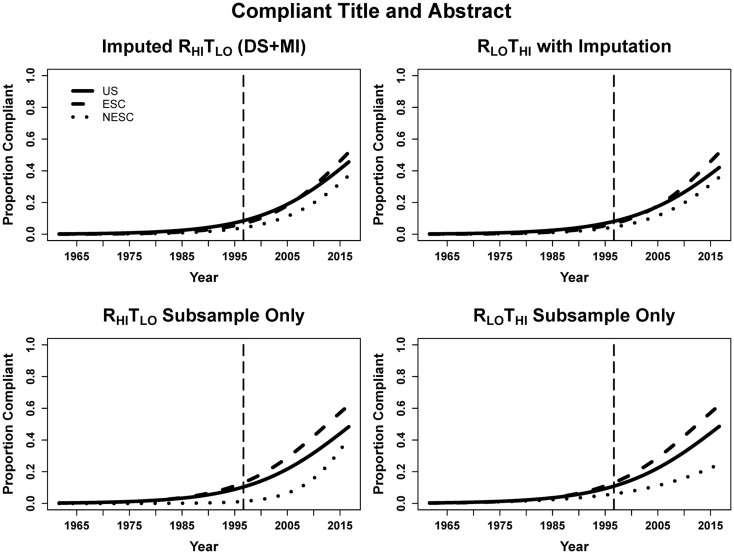
**Logistic curves for the combination of title and abstract compliance using imputation, the R_HI_T_LO_ and R_LO_T_HI_ for the large sample and subsample by year**. The vertical line represents the initiation of the CONSORT guidelines in 1996. US, United States; ESC, non-US but primarily English speaking countries; NESC, all other countries.

When looking at abstracts using the DS + MI estimates where both the title and abstract were compliant with CONSORT guidelines, entries published every year after CONSORT had 11.6% greater odds of being compliant. However, we see that at the time of CONSORT publication the NESC abstracts were significantly less compliant than US abstracts, but not ESC. Both ESC and NESC entries increased odds of compliance more rapidly than US (14.4% for ESC, 14.0% for NESC, and 11.6% for US, calculated by multiplying the year and respective interaction terms). Again, the small sample size resulted in only year being a significant predictor in the subsample (Table 6; Figure 5).

**Table 6:** Logistic regression of large sample and subsample for compliant titles and abstracts*.

	R_HI_T_LO_ imputed (DS + MI)^a^	R_LO_T_HI_ with imputed RCT^b^	R_HI_T_LO_ subsample	R_LO_T_HI_ subsample
Intercept	0.093 (0.028, 0.314); *p* = 0.001	0.089 (0.017, 0.470); *p* = 0.007	0.116 (0.062, 0.216); *p* < 0.001	0.122 (0.067, 0.225); *p* < 0.001
ESC	0.770 (0.349, 1.701); *p* = 0.499	0.804 (0.417, 1.552); *p* = 0.497	1.304 (0.462, 3.682); *p* = 0.617	1.237 (0.442, 3.465); *p* = 0.685
NESC	0.460 (0.257, 0.823); *p* = 0.011	0.536 (0.367, 0.783); *p* = 0.002	0.115 (0.007, 1.968); *p* = 0.135	0.523 (0.116, 2.359); *p* = 0.399
Year	1.116 (1.082, 1.151); *p* < 0.001	1.111 (1.086, 1.136); *p* < 0.001	1.110 (1.050, 1.174); *p* < 0.001	1.108 (1.049, 1.170); *p* < 0.001
ESC-by-year	1.026 (1.000, 1.052); *p* = 0.052	1.030 (1.010, 1.051); *p* = 0.005	1.014 (0.926, 1.112); *p* = 0.760	1.017 (0.928, 1.113); *p* = 0.722
NESC-by-year	1.021 (1.003, 1.040); *p* = 0.025	1.018 (1.002, 1.035); *p* = 0.026	1.098 (0.894, 1.350); *p* = 0.373	0.980 (0.866, 1.109); *p* = 0.749

*All values are presented as: “odds ratios (95% CI); p value.”

*^a^The higher rigor, lower throughput (R_HI_T_LO_) imputed values represent the double sampling with multiple imputation (DS + MI) approach*.

*^b^Values in the lower rigor, higher throughput (R_LO_T_HI_) with imputed RCT column represent the results of the R_LO_T_HI_ method of the entries in the large sample determined to be RCT based on the imputation*.

*ESC, non-US but primarily English speaking countries; NESC, all other countries*.

## General Discussion and Conclusion

Our results support our hypothesis that DS + MI would result in improved precision and accuracy of a large sample estimate. Using a double sampling approach with multiple imputation improved the precision of the point estimates compared to the subsample alone, as evidenced by the tightened confidence intervals around the logistic regression coefficients. We interpret the difference in point estimates between the R_LO_T_HI_ results and the DS + MI results, which are more similar to the subsample R_HI_T_LO_ results, to be evidence of improved accuracy. Specifically, when comparing the R_HI_T_LO_ to R_LO_T_HI_ methods, similar patterns were observed between the subsample and large sample in which R_HI_T_LO_ estimates that were higher than the R_LO_T_HI_ estimates in the subsample tended to be higher in the large sample, and vice versa. As expected, employing multiple imputation reduced the confidence intervals of those estimates compared to the subsample alone, improving precision. The reduced variance in estimates between the R_HI_T_LO_ and R_LO_T_HI_ models were not as dramatic as we had predicted because the R_LO_T_HI_ we employed was generally a highly correlated proxy for our R_HI_T_LO_ data, as evidenced by the corresponding phi coefficients. One would expect that with poorer correlation between R_LO_T_HI_ and R_HI_T_LO_, the information gained from DS + MI would improve precision considerably.

In our illustration, we were able to demonstrate that US had significantly higher reporting compliance before the implementation of the CONSORT guidelines relative to NESC but not ESC. However, over time, both ESC and NESC have been improving reporting compliance more rapidly than US abstracts, though it appears that compliance has increased over the years for all countries. Of the two criteria, we investigated structured abstract compliance was higher, which may be the result of journal requirements that submitted manuscripts contain structured abstracts (13). When examining the compliance of both the title and abstract together, compliance is lower. This suggests that authors are doing a better job of being compliant with the title or abstract but not both simultaneously.

In conclusion, using double sampling with multiple imputation allows for tractable large sample estimation for meta-research questions in situations where performing a comprehensive higher rigor, lower throughput evaluation on the entire corpus is impractical. This method is flexible and can be applied to many questions conditional on assumptions being met, namely that data are missing completely at random, expert ratings are sufficiently valid to be of intrinsic interest, that ratings comprise an exhaustive set of options, and sufficient data are collected to inform the imputation. In the presented example, the data were missing completely at random because we retrieved all available entries from PubMed based on the previously mentioned filters; expert ratings were conducted by trained, PhD-level scientists; the possible ratings for title and abstract compliance were exhaustive; and the sample size chosen for this study was appropriate to illustrate the feasibility of this method. One limitation of the present investigation is that R_HI_T_LO_ results were not obtained for an entire dataset, and thus we were only able to compare the results of the subsample and rely on established statistical theory for our inferences. A key limitation is that the method will only be useful when there is some imperfect correlation between the poorer and better measurement methods that is modeled effectively in the imputation process. To the extent that the relation approaches 0 or 1, the two-stage strategy will be of limited value. So too, if the functional form of the relation cannot be modeled properly in the imputation process, the two-stage strategy may yield biased results. It is possible that use of other imputation algorithms may yield different results. It is our hope that more researchers will continue to evaluate, validate, and extend the use of this method when conducting meta-research of the scientific literature.

## Author Contributions

PLC, AWB, JAD, and DBA made substantial contributions to the conception and design of this work, the analysis, and interpretation of the data; drafted this work and revised it critically for important intellectual content; provided final approval of the version to be published; and agree to be accountable for all aspects of this work in ensuring that questions related to the accuracy or integrity of any part of this work are appropriately investigated and resolved.

## Conflict of Interest Statement

The authors declare that the research was conducted in the absence of any commercial or financial relationships that could be construed as a potential conflict of interest.

## Supplementary Material

The Supplementary Material for this article can be found online at http://www.frontiersin.org/Journal/10.3389/fnut.2015.00006/abstract

## Abbreviations

CONSORT, consolidated standards of reporting trials; DP, date of publication; DS + MI, double sampling with multiple imputation; ESC, non-US but primarily English speaking countries; MCAR, missing completely at random; NESC, all other countries; PL, place of publication; RCT, randomized controlled trial; R_HI_T_LO_, higher rigor, lower throughput; R_LO_T_HI_, lower rigor, higher throughput.
