# Supplementary material for: Double Sampling with Multiple Imputation to Answer Large Sample Meta-Research Questions: Introduction and Illustration by Evaluating Adherence to Two Simple CONSORT Guidelines
Source: Front Nutr. 2015 Mar 9;2:6. doi: 10.3389/fnut.2015.00006 (PMC4428480; doi:10.3389/fnut.2015.00006)
Supplement: Supplementary file 1 [file Presentation_1.PDF]

Supplementary Material

Double sampling with multiple imputation to answer large sample meta-research questions: Introduction and illustration by evaluating adherence to two simple CONSORT guidelines

Patrice L. Capers[1], Andrew W. Brown[1], John A. Dawson[1,2], David B. Allison[1,2,3,4]*

[1]School of Public Health, Office of Energetics and Nutrition Obesity Research Center, University of Alabama at Birmingham, Birmingham, AL, USA
[2]Section on Statistical Genetics, University of Alabama at Birmingham, Birmingham, AL, USA
[3]Department of Nutrition Sciences, University of Alabama at Birmingham, Birmingham, AL, USA
[4]Department of Biostatistics, University of Alabama at Birmingham, Birmingham, AL, USA

*** Correspondence:** Dr. David B. Allison, University of Alabama at Birmingham, 1665 University Blvd, RPHB140J, Birmingham, AL 35294 – 0022.
dallison@uab.edu

1. Supplementary Data

2. Supplementary Figures and Tables

2.1. Supplementary Tables

Table S1. Words using the $R_{LO}T_{HI}$ method for structured abstracts.

Abstract heading	Words*
Introduction	Problem, context, rationale, background, purpose, objective, aims, introduction, hypothesis, goals
Methods	Design, methods, methodology, method, setting, participants, interventions, patients, subjects, outcome measures
Results	Results
Conclusion	Discussion, conclusion, clinical significance, level of evidence, interpretation

* The words used in the search were entered as regular expressions in R, with all search terms and abstract text changed to lower case. Search behavior in other systems (e.g., PubMed) may be different.

Table S2. Characterization of places published.

Group	Total	Subsample	Subsample (RCTs only)	Countries
United States (US)	164,491	257	209	United States
English Speaking Countries (ESC)	94,501	151	120	Canada<br>Ireland

				Jamaica<br>New Zealand<br>Australia<br>England<br>Wales<br>Scotland<br>Northern Ireland<br>Kenya<br>Malawi<br>Nigeria<br>Uganda<br>Zimbabwe
Others (NESC)	62,877	92	70	Argentina<br>Austria<br>Bangladesh<br>Belgium<br>Bosnia And Hercegovina<br>Brazil<br>Bulgaria<br>Chile<br>China<br>Colombia<br>Croatia<br>Czech Republic<br>Czechoslovakia<br>Denmark<br>Egypt<br>Ethiopia<br>Finland<br>France<br>Georgia (Republic)<br>Germany<br>Germany, East<br>Germany, West<br>Ghana<br>Greece<br>Hong Kong<br>Hungary<br>India<br>Indonesia<br>Iran<br>Israel<br>Italy<br>Japan<br>Korea<br>Korea (South)<br>Kuwait<br>Lebanon

				Lithuania<br>Luxembourg<br>Macedonia<br>Malaysia<br>Mexico<br>Nepal<br>Netherlands<br>Norway<br>Pakistan<br>Papua New Guinea<br>Peru<br>Philippines<br>Poland<br>Portugal<br>Puerto Rico<br>Romania<br>Russia (Federation)<br>Saudi Arabia<br>Senegal<br>Serbia<br>Serbia And Montenegro<br>Singapore<br>Slovakia<br>Slovenia<br>South Africa<br>Spain<br>Sri Lanka<br>Sweden<br>Switzerland<br>Taiwan<br>Tanzania<br>Thailand<br>Tunisia<br>Turkey<br>Ukraine<br>United Arab Emirates<br>Uruguay<br>Venezuela<br>Yugoslavia<br>Zambia
